# Supplementary material for: High-fat diets promote peritoneal inflammation and augment endometriosis-associated abdominal hyperalgesia
Source: Front Endocrinol (Lausanne). 2024 Mar 15;15:1336496. doi: 10.3389/fendo.2024.1336496 (PMC10978581; doi:10.3389/fendo.2024.1336496)
Supplement: Supplementary file 1 [file DataSheet_1.docx]

Supplementary Materials

# Supplementary Data sheet

## Supplementary Figures

## Supplementary Figure S1. Evaluation of time-dependent endometriosis-associated hyperalgesia after ELL induction.

(**A**) Abdominal retraction and (**B**) hind paw withdrawal thresholds were evaluated by the von Frey test with SD on Days -1, 3, 7, 14, 21, and 42 after ELL induction. Data are shown as mean ± SEM (n = 5 or 6). One-way ANOVA followed by Tukey’s post hoc test was used to compare the differences in abdominal and hind paw withdrawal thresholds across time points. a vs b; P<0.05. ELL: endometrial-like lesion.

## Supplementary Figure S2. Flow cytometer analysis for peritoneal immune cells at 12 weeks in the baseline study.

(**A**) Quantification of CD11b+ (MΦ), CD3+ (T-cells), CD19+ (B-cells), and Ly6C+ cells (n=5). (**B**) TIM4+ and Ly6C+ MΦ were quantified in the PF (n=5). Student t-test was used to compare the difference between SD and HFD groups (no difference was detected). SD: standard diets, HFD: high-fat diets, and MΦ, macrophages.

**Supplementary Figure S3. Immunostaining of pain-related markers in DRG.**

Representative single and merged staining images of neurofilament (green) and pain-related markers (red (**A**) BDNF, (**B**) CGRP, (**C**) SP, and (**D**) TRPV1) in DRG.

## Supplementary Table

**Supplementary Table S1**. Antibodies and Reagents for Flow Cytometry and Immunochemistry.

## Supplementary Method

**Supplementary Method.** Preparation of donor tissues in the mouse model of endometriosis.
