# Supplementary material for: High-fat diets promote peritoneal inflammation and augment endometriosis-associated abdominal hyperalgesia
Source: Front Endocrinol (Lausanne). 2024 Mar 15;15:1336496. doi: 10.3389/fendo.2024.1336496 (PMC10978581; doi:10.3389/fendo.2024.1336496)
Supplement: Supplementary file 2 [file DataSheet_2.docx]

*Supplementary Methods:*

To establish the mouse model of endometriosis, we first induced a “menses-like” event in donor mice. The donor female mice were ovariectomized and rested for 7-10 days to remove endogenous ovarian hormones. Then, mice were primed with 100 ng of estradiol-17β (E_2_) in corn oil for 3 days (days 1 to 3). For days 6, 7, and 8, mice were treated with 1 mg of progesterone (P_4_) and 5 ng of E_2_. On days 9 to 13, mice were treated with 1 mg of P_4_ daily. All hormone treatments were via subcutaneous (s.c.) injections. Decidualization was induced by delivering 20 μL of sesame oil using the mNSET™ (Non-Surgical Embryo Transfer) Device to the uterus on day 9. On day 13, “menses-like” endometrial tissue mass was scraped from the myometrium 4 hours after the last P_4_ injection. The collected tissue mass was then minced and suspended in 0.2 mL of sterilized PBS, passed through a 19-gauge needle, and injected intraperitoneally i.p. (50 mg per mouse) into naive recipient mice under anesthesia via inhaled isoflurane.
