# Supplementary figures and images for: High-fat diets promote peritoneal inflammation and augment endometriosis-associated abdominal hyperalgesia
Source: Front Endocrinol (Lausanne). 2024 Mar 15;15:1336496. doi: 10.3389/fendo.2024.1336496 (PMC10978581; doi:10.3389/fendo.2024.1336496)

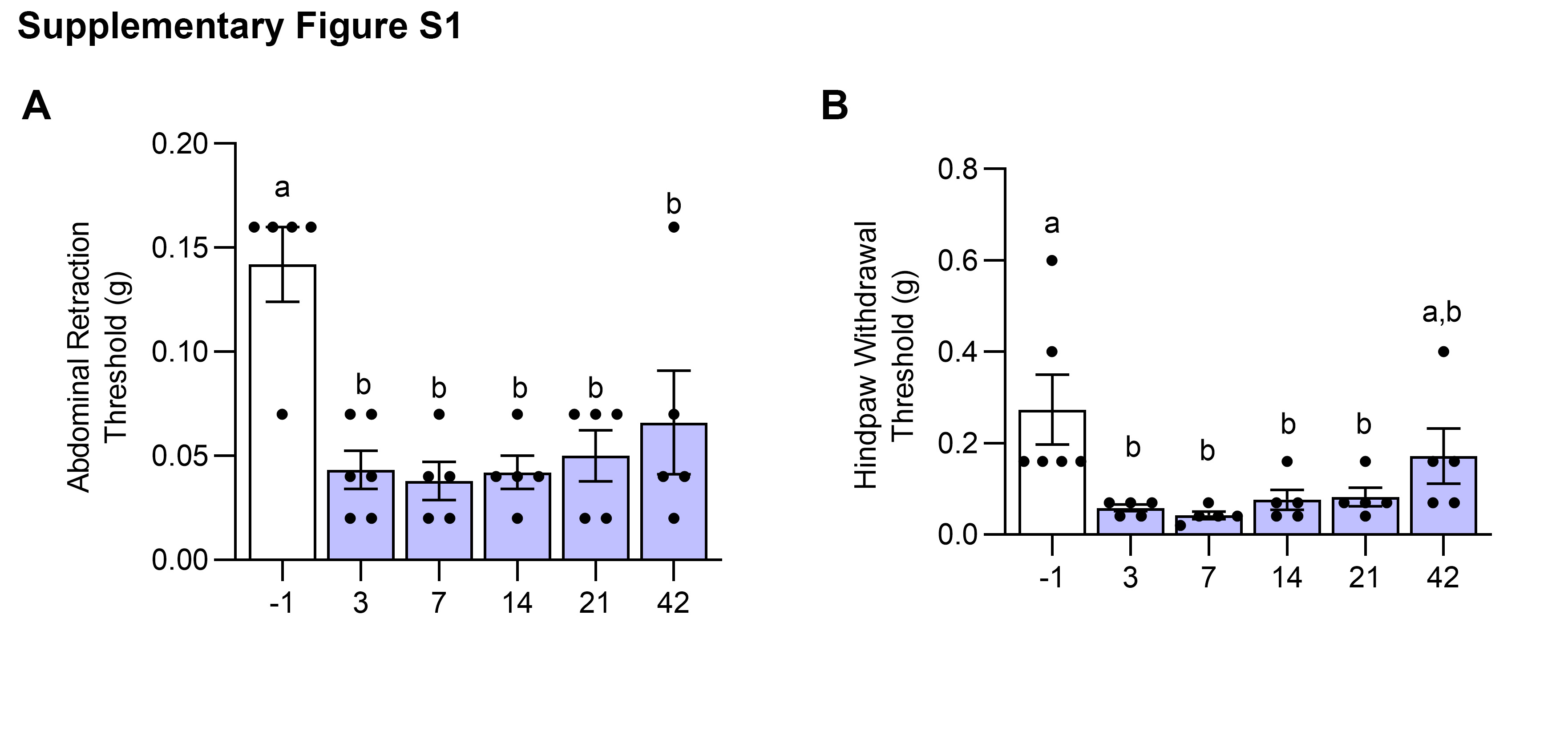

Supplement: Supplementary file 4 [file Image_1.jpg]

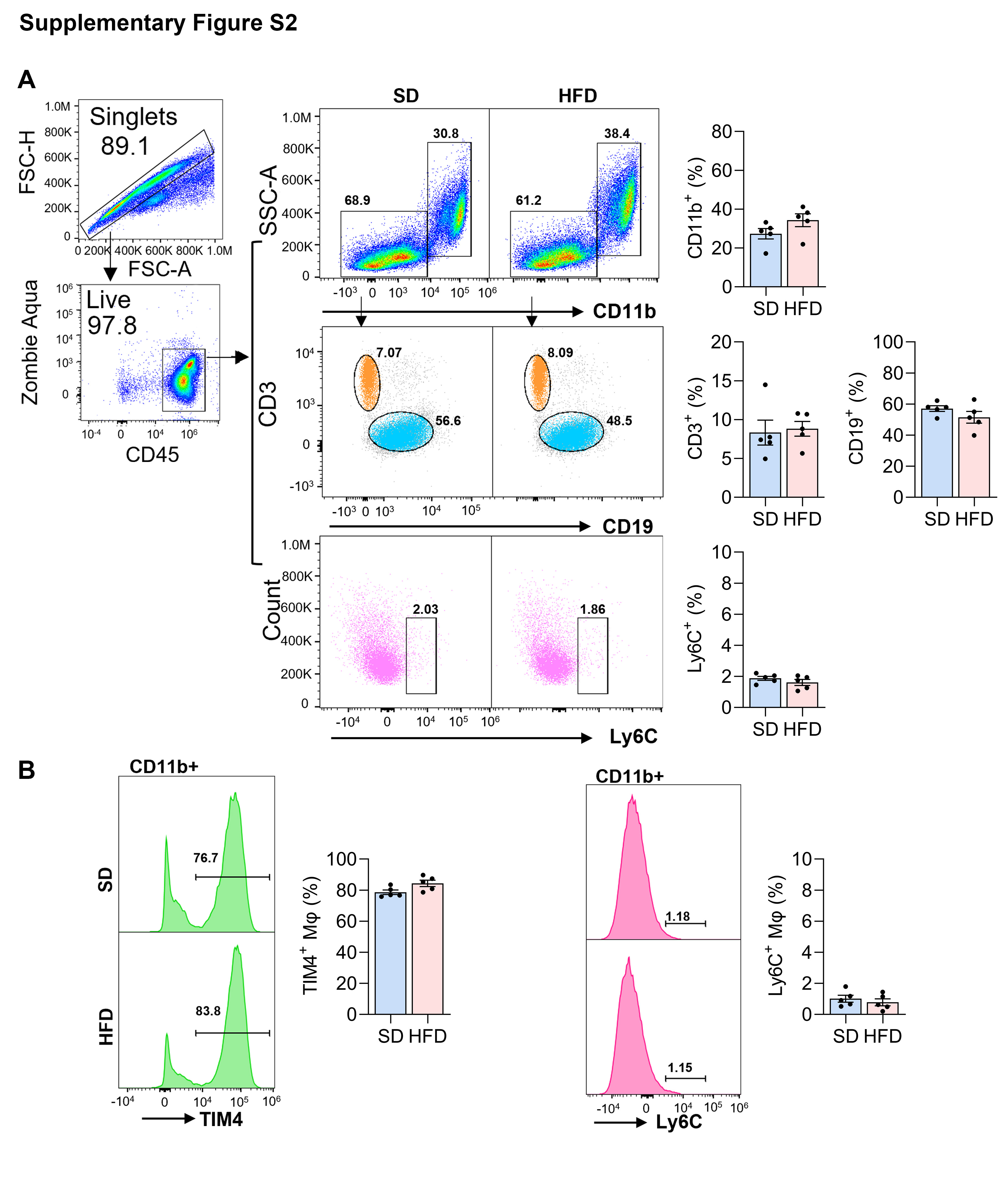

Supplement: Supplementary file 5 [file Image_2.jpg]

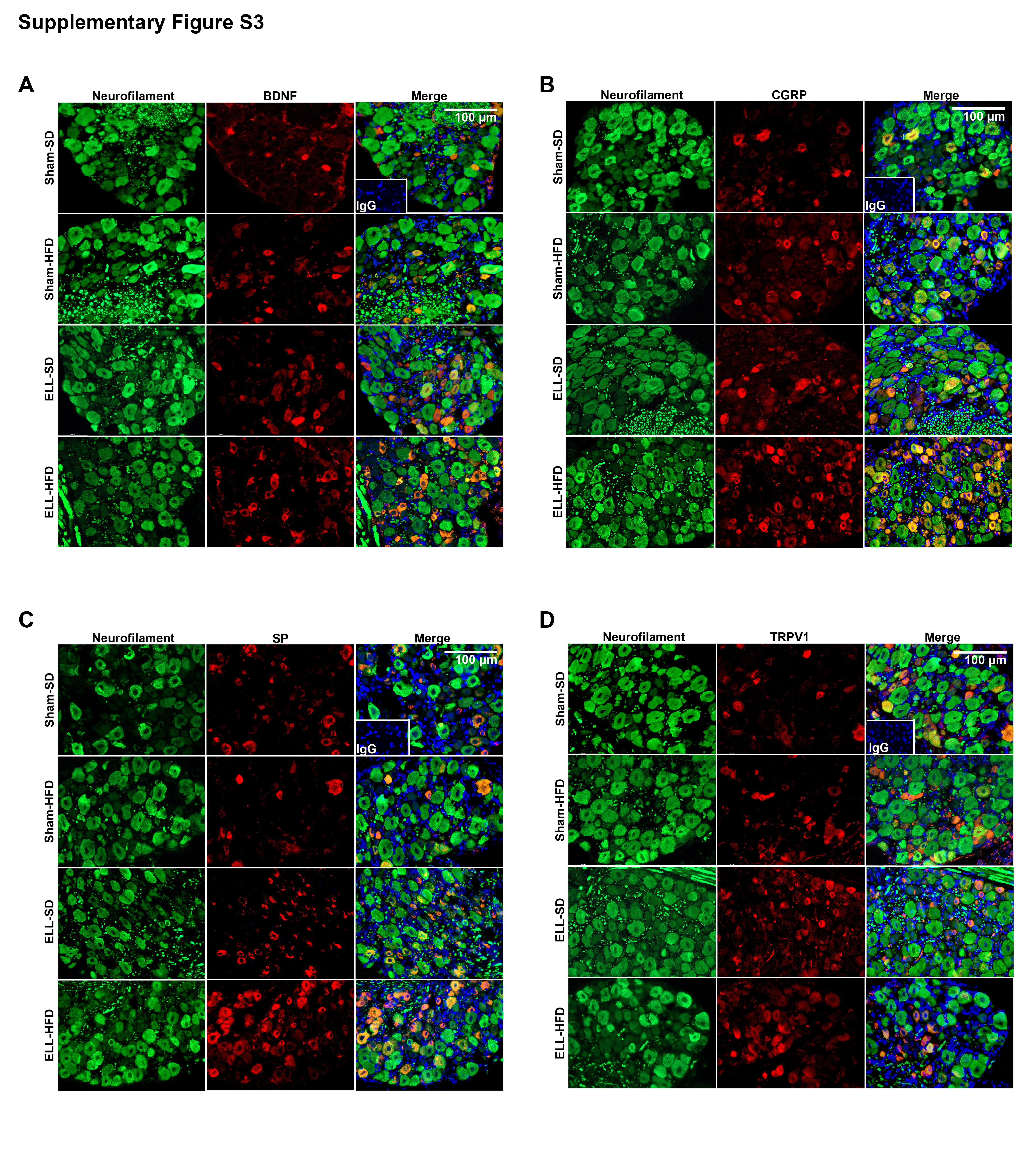

Supplement: Supplementary file 6 [file Image_3.jpg]
